# Supplementary material for: The taming of the weed: Developmental plasticity facilitated plant domestication
Source: PLoS One. 2023 Apr 7;18(4):e0284136. doi: 10.1371/journal.pone.0284136 (PMC10081796; doi:10.1371/journal.pone.0284136)
Supplement: Attachment — Submitted filename: Response to reviewers.docx [file pone.0284136.s002.docx]

Dear editors and reviewers,

We are very grateful to the reviewers for their kind and thoughtful comments. This work was very laborious, and it is satisfying to know that it is recognized as valuable despite its imperfections.

**Review 1**

1. *Regarding the evidence for genetic assimilation in maize domestication, the authors say that "this plasticity was lost during domestication because a) the environment was rendered more predictable by people, and; b) the expression of a teosinte-like phenotype was maladaptive in agroecosystems." Lorant et al. and Piperno et al. considered that genetic assimilation (GA) occurred in significant part because artificial selection was placed on the maize-like plastic teosinte phenotypes, as the phenotypes offered benefits to cultivators in way of ease of harvesting and other features, and would have been selected for. At least for maize and possibly other taxa where plasticity exists or may be found, the role of purposeful artificial selection should be emphasized more in the authors' discussion of how GA occurred.*

We rewrote this paragraph to make it clearer that we also think that human (artificial) selection (both purposeful and otherwise) played a role in genetic assimilation, and that there are different evolutionary circumstances that might lead to genetic assimilation, not just one.

2. *Also, there should be attention somewhere in the manuscript to the fact that environmental changes in the absence of human influences can cause plasticity in wild progenitors of crops; this effect would probably be more important in those crops domesticated shortly after the Pleistocene ended and appear to have operated in the teosinte to maize case.*

We agree, and we have added a few sentences calling attention to this scenario to *5. Discussion*. Hopefully our new paragraph in response to the previous critique will also help make this point, since we review hypothetical circumstances under which genetic assimilation might occur in nature with no input from people.

**Reviewer 2**

1. *To be clear, this study doesn’t demonstrate genetic assimilation in the same way as it shows how traits that are both adaptive to the plant and attractive to humans could arise through developmental plasticity in a very short time frame. Nor does this study claim to do so. But this research does lay-out a very plausible scenario as to how this could have happened in the case of erect knotweed. It is hoped that further experimental studies, coupled with genetic analysis, will provide this demonstration. And it would be good to hear more on how this line of inquiry might be pursued in the future.*

We added a discussion of how we might strengthen the case for domestication via genetic assimilation to *5. Discussion*. Erect knotweed has essentially no genetic resources (not even a reference genome) and its domesticated form is extinct, so it would be nearly impossible to *prove* that genetic assimilation occurred in this case. It might be possible (though very time consuming) to experimentally re-domesticate it, which might involve a canalization of currently plastic phenotypic characters. See revised manuscript for a longer discussion.

2. *I have very few suggestions for revision and believe that the manuscript could easily be published as is. I might, however, suggest a bit more on the earlier experimental work with this species that demonstrated the link between thinning plots of free-growing knotweed and changes in branch architecture and seed productivity. At the time this earlier work was done the impact of these activities on tubercle morph expression was unclear. Indeed, if I remember correctly, it seemed that the activities that encouraged the expression of these two traits that would have been attractive to human harvesters also encouraged the expression of tubercled morphs that would have inhibited the success of sown seeds in human tended garden plots. This study would seem to show that this is not the case and that these activities, coupled with later harvest schedules, would have favored seeds that would do well in such plots.*

Reviewer 2 remembers correctly, and we did have a brief discussion of this in our hypotheses section. Those ideas were based on Sonia Sultan’s results with a closely related species, which inspired two of the 4 experiments that we are drawing on for this paper. We should have made this clearer, so we added a longer discussion of this topic to section *2.4: Erect Knotweed Case Study.* We also added a second sentence clarifying and reinforcing Mueller’s previous experimental results regarding plant density and yield to this section, and brought this result up again in the *5. Discussion.*

3. *At several points in the manuscript the authors refer to Table 1 when I think they meant to refer to Table 2.*

Thanks for catching this. We checked all references to Tables and Figures and insured that they are correct.

4. *The density column in Table 2 could use with a bit more explanation. I’m not sure what the [53] figures in a number of cells mean.*

We added a sentence clarifying the plant density column to *3. Materials and Methods.* I don’t understand what the second half of this comment is referring to: there are not [53]s in my version of this table.

5. *Figures showing the plants could also use a bit more explication in the heading to help the reader not familiar with growing knotweed (which I think would apply to a lot of folks) decipher what is being shown.*

I am not sure what additional information to include in the captions for these figures (maybe I am TOO familiar with growing erect knotweed to spot what is missing!), but I am happy to elaborate further with additional guidance.

6. *Not sure if underlying data are presented here as I don’t see that there is supplemental information included with the paper.*

We have uploaded the underlying data as Supplemental Information.

Please let us know if you would like us to make any further changes to this manuscript. As usual, we believe that responding to these critiques has made this a better paper.

Sincerely,

Natalie G. Mueller

Assistant Professor of Anthropology

Washington University in St. Louis
